# Implication of DNA Demethylation and Bivalent Histone Modification for Selective Gene Regulation in Mouse Primordial Germ Cells

**DOI:** 10.1371/journal.pone.0046036

**Published:** 2012-09-28

**Authors:** Kentaro Mochizuki, Makoto Tachibana, Mitinori Saitou, Yuko Tokitake, Yasuhisa Matsui

**Affiliations:** 1 Cell Resource Center for Biomedical Research, Institute of Development, Aging and Cancer, Tohoku University, Sendai, Japan; 2 Experimental Research Center for Infectious Diseases, Institute for Virus Research, Kyoto University, Kyoto, Japan; 3 Department of Anatomy and Cell Biology, Graduate School of Medicine, Kyoto University, Kyoto, Japan; 4 JST, CREST/ERATO, Kyoto, Japan; Wellcome Trust Centre for Stem Cell Research, United Kingdom

## Abstract

Primordial germ cells (PGCs) sequentially induce specific genes required for their development. We focused on epigenetic changes that regulate PGC-specific gene expression. *mil-1*, *Blimp1*, and *Stella* are preferentially expressed in PGCs, and their expression is upregulated during PGC differentiation. Here, we first determined DNA methylation status of *mil-1*, *Blimp1*, and *Stella* regulatory regions in epiblast and in PGCs, and found that they were hypomethylated in differentiating PGCs after E9.0, in which those genes were highly expressed. We used siRNA to inhibit a maintenance DNA methyltransferase, *Dnmt1*, in embryonic stem (ES) cells and found that the flanking regions of all three genes became hypomethylated and that expression of each gene increased 1.5- to 3-fold. In addition, we also found 1.5- to 5-fold increase of the PGC genes in the PGCLCs (PGC-like cells) induced form ES cells by knockdown of *Dnmt1*. We also obtained evidence showing that methylation of the regulatory region of *mil-1* resulted in 2.5-fold decrease in expression in a reporter assay. Together, these results suggested that DNA demethylation does not play a major role on initial activation of the PGC genes in the nascent PGCs but contributed to enhancement of their expression in PGCs after E9.0. However, we also found that repression of representative somatic genes, *Hoxa1* and *Hoxb1*, and a tissue-specific gene, *Gfap*, in PGCs was not dependent on DNA methylation; their flanking regions were hypomethylated, but their expression was not observed in PGCs at E13.5. Their promoter regions showed the bivalent histone modification in PGCs, that may be involved in repression of their expression. Our results indicated that epigenetic status of PGC genes and of somatic genes in PGCs were distinct, and suggested contribution of epigenetic mechanisms in regulation of the expression of a specific gene set in PGCs.

## Introduction

Germ cells are the only cells capable of giving rise to truly totipotent cells, via differentiation to sperms/eggs and subsequent fertilization. In mouse embryos at around embryonic day (E) 7.25, a small population of primordial germ cells (PGCs) in the extraembryonic mesoderm and derived from the epiblasts is first “fate-determined”. Shortly before PGC fate determination, cell type-specific expression of Blimp1/Prdm1 and Prdm14 initiates in PGC precursors; these proteins are key transcriptional regulators of PGC development. Blimp1/Prdm1 and Prdm14 repress the somatic mesodermal program [1]–[3]. Furthermore, many pluripotency-related genes, including *Oct4*, *Nanog*, and *Sox2*, are expressed specifically in PGCs [2], [4]. Oct4 plays essential roles in PGCs fate determination [5]. Once fate-determined, PGCs start to migrate to genital ridges, the future gonads; there, they rapidly proliferate and increase in number. A portion of PGCs is eliminated by apoptosis during this period [6]. Nanos3 [7], [8] is initially expressed specifically in PGCs at around PGC fate determination, and it supports survival of migrating PGCs along with Oct4 [9] and Nanog [10], [11].

Genome-wide epigenetic changes also occur in migrating PGCs [summarized in 12], and Prdm14 is involved in this process [3]. After arrival at genital ridges, PGCs undergo further epigenetic changes such as DNA demethylation of imprinted genes and the repetitive sequences [13]. Germ cells thereafter stop proliferation at E14.5, and male germ cells are arrested in G1 phase of cell-cycle, resume proliferation at the time of birth as spermatogonial stem cells and part of them start to differentiate towards spermatozoa. At E14.5, female germ cells immediately enter meiosis, are soon arrested at meiotic prophase I. A part of oocytes then resumes meiosis according to estrus cycle in adult ovary and undergo further maturation. PGC-specific expression of the mouse Vasa homolog (Mvh/Ddx4) and Dazl (Daz-like) is initiated in differentiating PGCs at E11.5 [14], and these genes are required for progression through meiotic prophase I in male germ cells [15] and for sex-specific differentiation of fetal germ cells [16], [17], respectively. In addition, Nanos2 is specifically expressed in male PGCs after PGC colonization of the genital ridges, and it suppresses meiosis and promotes male germ cell differentiation [7], [18]. Furthermore, our previous investigation revealed that the meiosis-specific histone methyltransferase Meisetz/Prdm9 is specifically expressed in early meiotic germ cells both in testes and ovaries and plays an essential role in proper progression through early meiotic prophase [19]. Over the course of several developmental stages, PGCs sequentially induce many specific genes that are required for the proper progression of multiple unique developmental events [summarized in 20]; therefore, it is important to elucidate the mechanism that control PGC-specific gene expression to understand regulation of PGC development.

Several experiments have been performed to identify *cis*-regulatory elements within the flanking regions of PGC genes using transgenic mice carrying reporter genes, such as *lacZ* and *green fluorescent protein* (*GFP*), fused to these flanking regions. For example, 18.0 kbp of the flanking sequences of *Oct4* gene is sufficient for reproducing the endogenous *Oct4* expression pattern, i.e. specific expression in blastomere and inner cell mass in pre-implantation embryos, and in epiblast and PGC after implantation in transgenic mice [21], [22]. Within this 18.0 kbp region, the proximal enhancer (PE), which is located 1.4 kbp to 0.3 kbp upstream from a transcription start site (TSS), directs epiblast-specific expression, whereas the distal enhancer (DE), located 4.6 kbp to 2.0 kbp upstream from a TSS, is necessary for expression in PGCs [21], [22]. In addition, *mil-1* (*fragilis/Ifitm3*), is a representative PGC gene that is first expressed in PGC precursors [23], [24]. A 3.0 kbp sequence in the 5′-flanking region of *mil-1* is necessary for PGC-specific expression at the time of their specification onward, and the *cis*-regulatory element, *Ifitm* genes consensus element (ICE) was particularly important for its PGC-specific expression. ICE is approximately 190 bp in length and contains a 90 bp short interspersed transposable element (SINE)-like sequence that is located at 2 kbp upstream from a TSS. ICE consensus sequences were also found within regions flanking other PGC genes [25]. Similarly, reporter constructions of other PGC genes (e.g. *Blimp1*, *Prdm14*, *Stella*/*Dppa3*, *Mvh*, and *Dazl*), mimicked the endogenous expression patterns in transgenic mice [1], [3], [26]–[28], but the critical *cis*-regulatory sequences in these constructs have not been identified yet. The molecular mechanisms controlling PGC-specific gene expression have been rarely studied; nevertheless, it is clear that some PGC-specific genes (e.g. *Mvh* and *Dazl*) are initially expressed after PGC colonize the genital ridges and that the regions flanking these genes are concomitantly demethylated in PGCs [14].

Epigenetic mechanisms are also involved in repressing expression of PGC genes in somatic cells. The repressive transcription factor E2F6 may be necessary to silence several PGC genes in somatic cells via DNA hypermethylation that locks the target promoters in transcriptionally inactive states [29]–[31]. In addition, suppression of *Oct4* expression in somatic cells by an orphan nuclear receptor, germ cell nuclear factor (GCNF), depends on DNA hypermethylation of the *Oct4* flanking region [32], [33]. Interestingly, in various types of human tumors, many testis-specific genes and PGC-specific genes are ectopically expressed, and CpG in the flanking regions are CpG-hypomethylated [34], [35]. Reportedly, the flanking regions of PGC-specific genes (e.g. *VASA* and *SCP1*/*SYCP1*) are generally CpG-hypermethylated in normal somatic tissues; these findings indicate that DNA demethylation activates ectopic expression in tumors [36]–[39]. Taken together, these findings indicate that DNA methylation prevents ectopic expression of PGC-specific and/or pluripotent-related genes in normal somatic cells. In addition, genome-wide DNA methylation analysis revealed that DNA methylation targeted to repress the germ cell related genes in pre- and post-implantation epiblast [40]; therefore, it is likely that there are epigenetic activating mechanisms that induce normal expression of specific genes in PGCs.

Here, we focused on detailed epigenetic changes of representative genes preferentially expressed in PGCs and somatic genes, and a possible role of DNA demethylation in the expression of PGC genes that are initially expressed around the time of PGC fate determination was also investigated. Our findings indicated that the regions flanking PGC genes that contain the consensus element, ICE, commonly underwent DNA demethylated in differentiating PGCs after E9.0 in which the expression of those genes was upregulated. We also showed that repression of the *Hox* genes, representative somatic genes, as well as a neural cell-specific *Gfap* gene in PGCs was not dependent on DNA methylation, but may be regulated by the bivalent histone modification.

## Results

### *mil-1* Regulatory Regions were Hypomethylated in Differentiating PGCs

We previously reported that 3.0 kbp of the 5′-flanking region of *mil-1* gene was necessary for PGC-specific expression [25], but the mechanisms that confer PGC-specific expression are not fully characterized. DNA methylation is one of the most well-known epigenetic mechanisms regulating gene expression, and methylation of CpG sites often represses gene expression. There are many CpG sites in the *mil-1* regulatory region; therefore, we first investigated the possible involvement of DNA demethylation in PGC-specific expression of *mil-1*.

**Figure 1:**
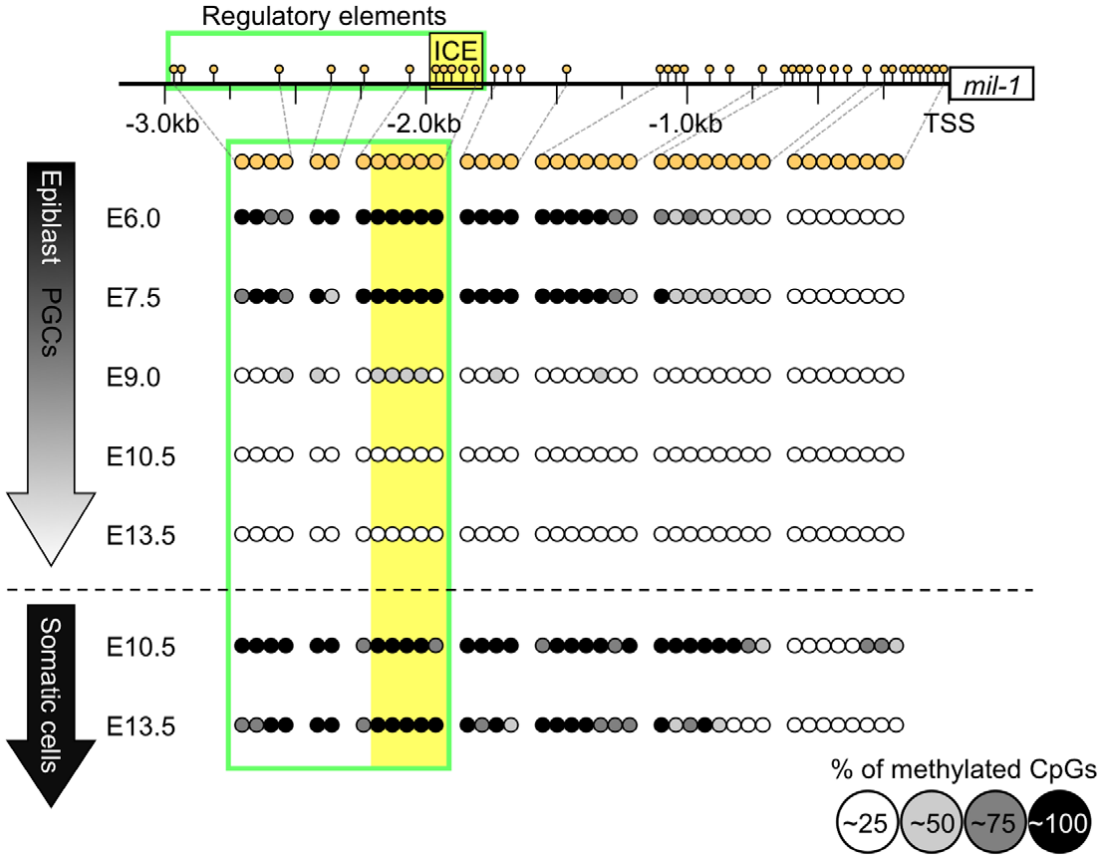
The regulatory region of *mil-1* becomes hypomethylated during PGC development. Bisulfite sequencing analysis of the regulatory region of *mil-1* was performed using epiblasts, PGCs/somatic cells purified as GFP positive/negative cells from embryos at each embryonic day (E). The rectangle containing *mil-1* in the top line represents an exon, and the numbers with ‘kb (kilobase)’ under the line indicate distance from the transcription start site (TSS). The box outlined in green represents the regulatory element required for PGC-specific expression and the *Ifitm* genes consensus element (ICE) is shown in more detail in Figure 4A. Each circle corresponds to a CpG site in the regulatory region, and the degree of gray in each circle corresponds to the level of DNA methylation.

**Figure 2:**
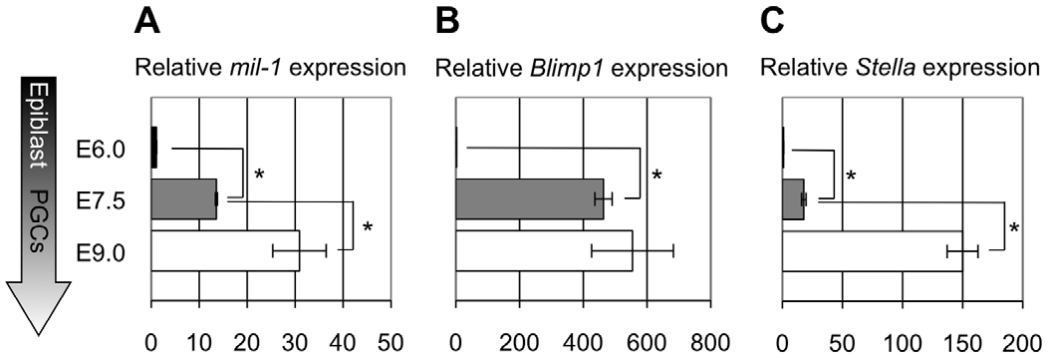
The expression of *mil-1*, *Blimp1*, and *Stella* become upregulated during PGC development. (A, B, C) Quantitative RT-PCR analysis of the expression of (A) *mil-1*, (B) *Blimp1*, and (C) *Stella* was performed using epiblasts (E6.0) and PGCs (E7.5 and E9.0). Histograms represent relative expression levels of these three genes at each developmental stage. The averages of expression levels in the epiblasts (E6.0) were set as 1.0. *Gapdh* PCR signal was used as an internal control to measure relative. The data were obtained from three individual embryos. *p<0.05. Error bars represent SEM.

To examine DNA methylation status of the *mil-1* regulatory region, bisulfite sequencing analysis was performed using epiblasts or PGCs and somatic cells purified as GFP-positive or GFP-negative cells, respectively, from the *Blimp1*- or *Oct4ΔPE*-GFP transgenic embryos at various developmental stages (Figure 1, S1). We found that the region near the transcription start site (TSS) was hypomethylated in all cell types tested, but the upstream regulatory region was hypermethylated (about 15% of CpGs on average in the regulatory element was demethylated) in the region of epiblast proximal to the adjacent extraembryonic ectoderm at E6.0 before any *mil-1* expression was evident (Figure 1, S1, Figure 2A) and in nascent PGCs at E7.5 just as *mil-1* expression was evident (Figure 1, S1, Figure 2A). The *mil-1* regulatory region was massively demethylated in migrating PGCs at E9.0 (Figure 1, S1, about 75% of CpGs on average in the regulatory element was demethylated), and finally became almost completely unmethylated in gonadal PGCs by E10.5 or E13.5 (Figure 1, S1, about 100% of CpGs on average in the regulatory element was demethylated). In contrast, the *mil-1* regulatory region remained hypermethylated in the surrounding somatic cells in fetal gonads, in which *mil-1* is hardly expressed [23] (Figure 1, Figure S1). Interestingly, the massive DNA demethylation of the regulatory region, that occurred between E7.5 and E9.0 in PGCs, was correlated with 2-fold upregulation of *mil-1* expression at this stage (Figure 2A). Based on these results, it was likely that DNA demethylation of the regulatory region of *mil-1* did not play a major role on initial activation of *mil-1* at the time of PGC-specification, but made a contribution to enhancement of *mil-1* expression after E7.5.

### DNA Demethylation Upregulates the *mil-1* Expression in ES Cells

**Figure 3:**
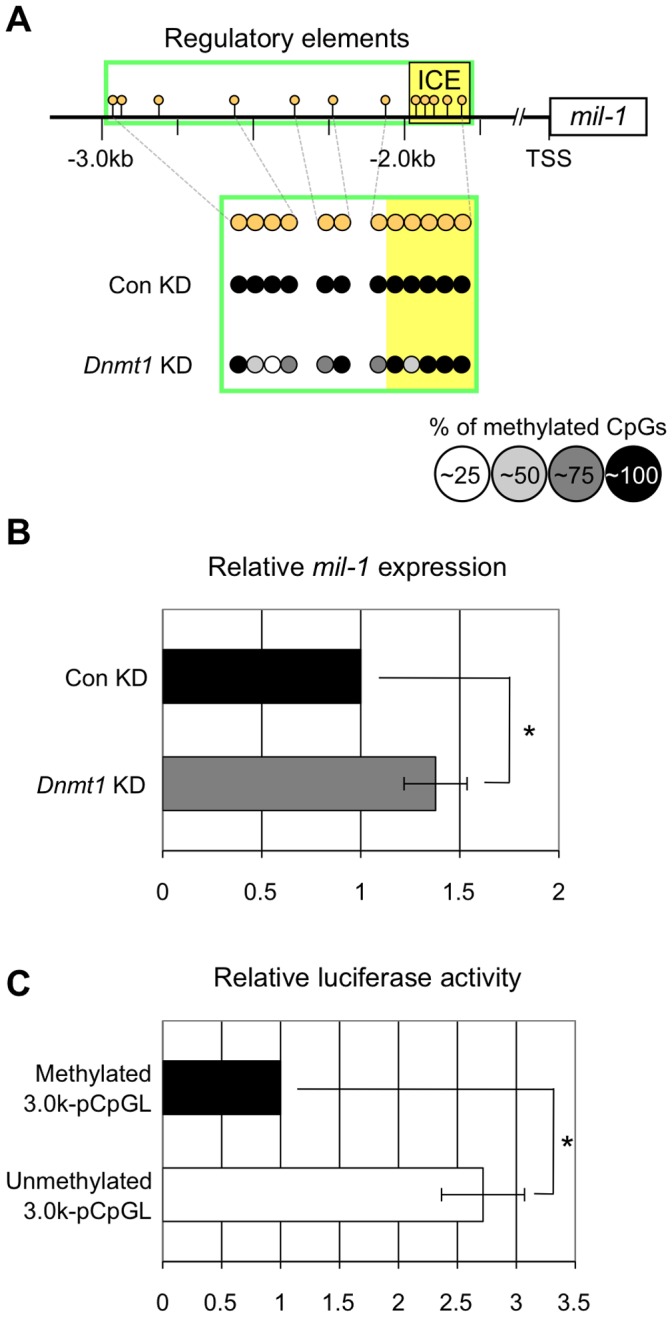
DNA demethylation of the regulatory region of *mil-1* resulted in upregulation of its expression in ES cells. (A) Bisulfite sequencing analysis of the regulatory region of *mil-1* and (B) quantitative RT-PCR analysis of *mil-1* expression were performed on embryonic stem (ES) cells with or without siRNA-mediates *Dnmt1* knockdown (*Dnmt1* KD/Con KD). (A) The regulatory region became more hypomethylated following *Dnmt1* knockdown. (B) Histogram represents the relative expression level of *mil-1* in the *Dnmt1*-knockdown ES cells. The expression level in the control ES cells (Con KD) was set as 1.0. *Gapdh* PCR signal was used an internal control to measure relative expression. The data were obtained from four independent experiments. *p<0.05. Error bars represent SEM. (C) Luciferase activity of the reporter vectors with methylated or unmethylated regulatory region of *mil-1* in ES cells. Luciferase activity was normalized against the activity of a cotransfected *Renilla* construct. The liciferase activity of the methylated construct (Methylated 3.0k-pCpGL) was set as 1.0. The data were obtained from six independent experiments. *p<0.05. Error bars represent SEM.

To evaluate role of DNA demethylation in regulation of *mil-1* expression, we knocked down *Dnmt1* in ES cells. Because *Dnmt1* is a maintenance DNA methyltransferase, DNA demethylation that is dependent on DNA replication should occur owing to the failure of DNA methylation maintenance in the knockdown cells. ES cells are pluripotent stem cells that partly share similar cellular characteristics with epiblasts, and the *mil-1* regulatory region was hypermethylated (Figure 3A, Con KD, Figure S3, about 5% of CpGs on average in the regulatory element was demethylated) and *mil-1* expression was relatively low in ES cells compared to that in PGCs [41]. Therefore, we predicted that the induction of *mil-1* expression in ES cells by forced DNA demethylation by *Dnmt1* knockdown likely mimicked that in PGCs during their determination. We transfected siRNA targeted for *Dnmt1* into undifferentiated ES cells and allowed the cell to undergo several rounds of DNA replication in culture for 72 hours, then we carried out bisulfite sequencing analysis of the *mil-1* regulatory region. As expected, the regulatory region became more hypomethylated in *Dnmt1*-knockdown ES cells (about 30% of CpGs on average in the regulatory element was demethylated) than in control ES cells (Figure 3A, *Dnmt1* KD, Figure S3). We next assessed levels of *mil-1* expression using quantitative RT-PCR analysis and confirmed that *mil-1* expression was about 1.5-fold higher in the knockdown ES cells than in the control ES cells (Figure 3B). We also found that DNA methylation of the *mil-1* regulatory region resulted in about 2.5-fold decrease of the expression of the luciferase reporter in ES cells (Figure 3C). These results suggested that DNA demethylation of the *mil-1* regulatory region resulted in upregulation of *mil-1* expression in ES cells.

### Regions Flanking Other PGC-specific Genes are also Demethylated during PGC Development

**Figure 4:**
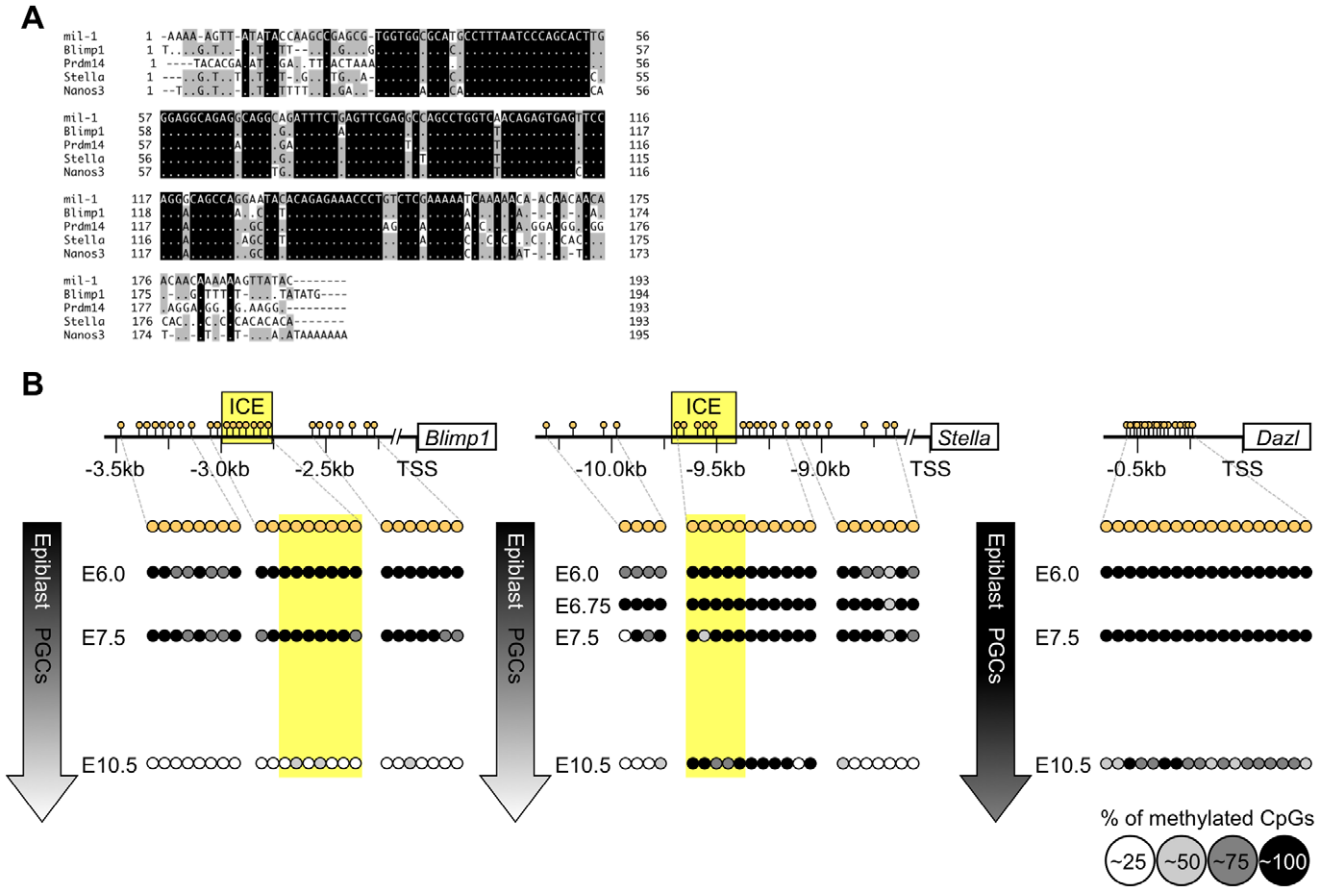
Flanking regions of the PGC-specific genes also become hypomethylated during PGC development. (A) Comparison of the *Ifitm* genes consensus element (ICE) of *mil-1*/*Ifitm3* [25] with the homologous sequences found in the putative regulatory regions flanking *Blimp1*/*Prdm1*, *Prdm14*, *Stella*/*Dppa3*, and *Nanos3*. (B) Bisulfite sequencing analysis of the flanking regions of *Blimp1*, *Stella*, and *Dazl* was performed using epiblasts (E6.0 and E6.75) and PGCs (E7.5 and E10.5). The flanking regions of *Blimp1* and *Stella*, like those in *mil-1*, were also progressively demethylated during PGC development, whereas that of *Dazl* was maintained hypermethylated in PGCs at E10.5.

The *cis*-regulatory element ICE that resides within the regulatory region of *mil-1* [25] was also found in the putative regulatory regions flanking some PGC genes including, but not limited to, *Blimp1*/*Prdm1*, *Prdm14*, *Stella*/PGC7/*Dppa3*, and *Nanos3* (Figure 4A). These findings indicated that ICE-associated regulatory mechanisms, including DNA demethylation of ICE or of its flanking sequences, may generally mediate the expression of those genes in PGCs. We analyzed *Blimp1* and *Stella* to investigate this possibility. Expression of *Blimp1* (a key transcriptional regulator for fate determination of PGCs) and of *Stella* (a marker of PGCs) starts in PGC precursors at E6.25 and in nascent PGCs at E7.0, respectively, and their expression is maintained in PGCs in fetal gonads at E13.5 (Figure 2B, 2C **)** [1], [24], [42], [43]. ICEs are located in putative regulatory regions of *Blimp1* and *Stella* at 3.0 and 9.5 kbp upstream, respectively, from the TSSs (Figure 4B, S2).

We examined DNA methylation status of the 5′-flanking regions, including the ICEs, of *Blimp1* and *Stella* in epiblasts and PGCs (Figure 4B, S2) and found that the flanking regions of *Blimp1* and *Stella* were hypermethylated in the E6.0 proximal epiblasts and in E6.75 posterior proximal epiblasts, respectively, and in the nascent PGCs at E7.5 (Figure 4B, S2, about 15% and 20% of CpGs on average in the flanking regions of *Blimp1* and *Stella*, respectively, were demethylated at E7.5). Subsequently, the flanking regions were demethylated in migrating PGCs by E10.5 (Figure 4B, S2, about 95% and 60% of CpGs on average in the flanking regions of *Blimp1* and *Stella*, respectively, were demethylated). Notably, about 1.2-fold and 8-fold upregulation of *Blimp1* and *Stella* expression, respectively, were observed between E7.5 and E9.0 when demethylation of their flanking regions were thought to be in progress (Figure 2B, 2C). Although upregulation of *Blimp1* was subtle, upregulation of *Stella* during this period was more evident (Figure 2). Therefore it was likely that DNA demethylation was not involved in the initial induction of those genes at PGC specification, but contributed to enhancement of *Stella* after E7.5. On the other hand, we cannot exclude a possibility that the tested flanking regions were not relevant to regulation of those genes and did not correctly reflect correlation of their DNA methylation status and expression.

We also examined DNA methylation status of the flanking region of another PGC-specific gene, *Dazl*, whose expression is upregulated at E11.5 [44], and found that it was maintained as more hypermethylated status compared with the regulatory regions of *mil-1*, *Blimp1* and *Stella* until E10.5 (Figure 4B, S2). The results showed that the timing of upregulation of the PGC gene expression was correlated to the timing of DNA demethylation of their regulatory regions.

### DNA Demethylation Upregulates Expression of *Blimp1* and *Stella* in ES Cells

**Figure 5:**
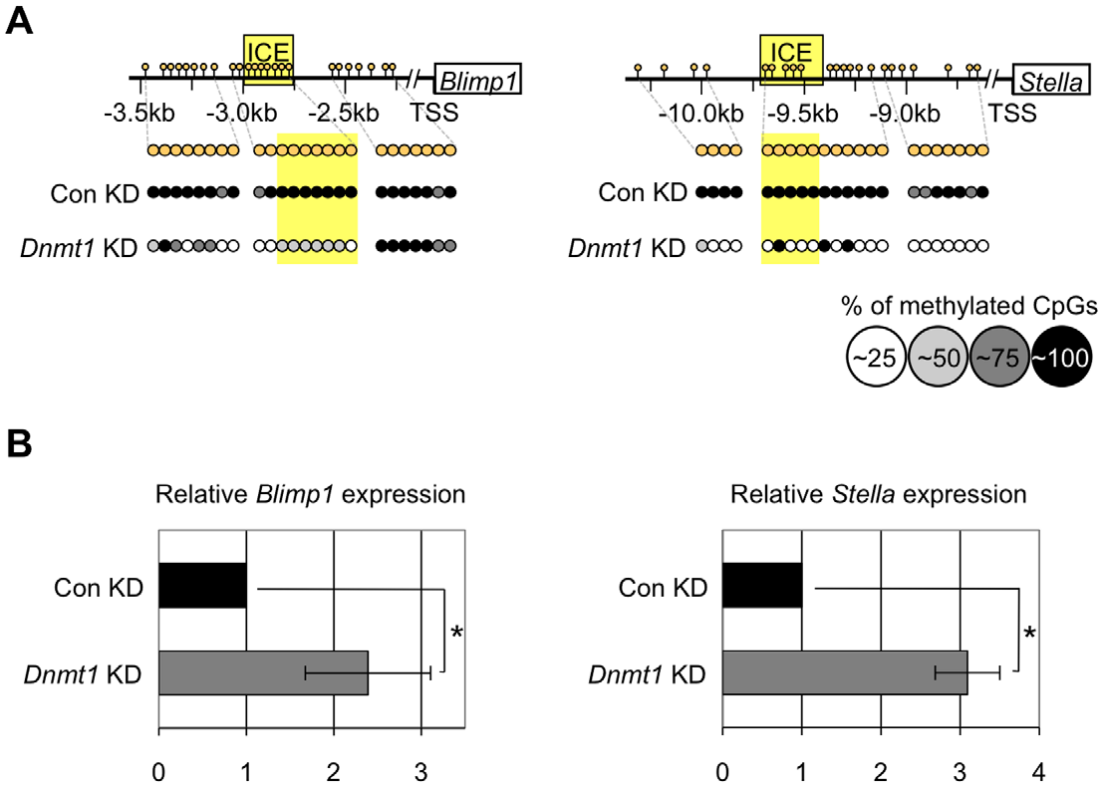
Knockdown of *Dnmt1* causes hypomethylation of *Blimp1* and *Stella* flanking regions and upregulation of *Blimp1* and *Stella* expression in ES cells. (A) Bisulfite sequencing analysis of the flanking regions of *Blimp1* and *Stella* and (B) quantitative RT-PCR analysis of *Blimp1* and *Stella* expression were performed using ES cells with or without *Dnmt1* knockdown treatment (*Dnmt1* KD/Con KD).

To evaluate the role(s) of DNA demethylation in expression of *Blimp1* and *Stella*, we knocked down *Dnmt1* in ES cells using siRNA. In undifferentiated ES cells, regions flanking *Blimp1* and *Stella* were hypermethylated (Figure 5A, Con KD, Figure S3, about 5% of CpGs on average in the flanking regions of *Blimp1* and *Stella* were demethylated). As expected, in the *Dnmt1*-knockdown ES cells, regions flanking *Blimp1* and *Stella* became more hypomethylated than in control ES cells (Figure 5A, *Dnmt1* KD, Figure S3, about 45% and 80% of CpGs on average in the flanking regions of *Blimp1* and *Stella*, respectively, were demethylated), and the levels of *Blimp1* and *Stella* expression were 2-fold and 3-fold higher (Figure 5B) than in the control ES cells. Although the effect of *Dnmt1* knockdown on demethylation of the flanking region of *Blimp1* and its upregulation was subtle, increased expression of *Stella* by *Dnmt1* knockdown was more evident. In PGCs, the flanking region of *Stella* was massively demethylated between E7.5 and E10.5 and its expression increased 8-fold between E7.5 and E9.0 (Figure 2). In addition, a previous study suggested possible involvement of DNA demethylation of *Stella* in its upregulation in pluripotential stem cells [45]. These results suggested that DNA demethylation of regions flanking *Stella* is involved in its upregulation in ES cells and in PGCs after E7.5.

As controls, we investigated the expression of *Oct3/4* and *Nanog* in *Dnmt1*-knochdown ES cells and their expression was not significantly affected by the knockdown of *Dnmt1* (Figure S4), indicating that the increased expression of the PGC-specific genes by knockdown of *Dnmt1* was not a consequence of differentiation of the pluripotential cells.

### The Expression of the PGC-specific Genes in the PGC-like Cells are Enhanced by Knockdown of *Dnmt1*

**Figure 6:**
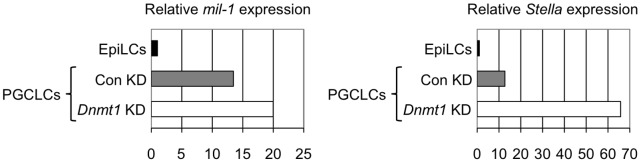
Knockdown of *Dnmt1* causes upregulation of *mil-1* and *Stella* expression in PGCLCs. Quantitative RT-PCR analysis of *mil-1* and *Stella* expression in PGCLCs (PGC-like cells) with or without *Dnmt1* knockdown (*Dnmt1* KD/Con KD). The expression level in EpiLCs was set as 1.0. Shown is a representative data from two independent experiments.

We further evaluated the functions of *Dnmt1* on regulation of PGC-specific gene expression in the PGC-like cells (PGCLCs) induced from ES cells in culture. As recently reported, ES cells differentiate to epiblast-like cells (EpiLCs) in the presence of ActivinA, bFGF and 1% Knock-Out Serum Replacement (KSR), and EpiLCs further give rise to PGCLCs when they are cultured with BMP4, LIF, SCF and EGF [46]. By using this culture, we knocked down *Dnmt1* in EpiLCs, and induced PGCLC after culturing for one more day. We quantified the expression of *mil-1* and *Stella* at the time of transfection of siRNA of *Dnmt1* and after differentiation to PGCLCs (Figure 6), and found that the expression of both genes in PGCLCs was 1.5-fold and 5-fold, respectively, increased by *Dnmt1* knockdown. In this experiment, it is likely that knockdown of *Dnmt1* just before or at the time of PGCLC induction resulted in increased expression of *mil-1* and *Stella*. Because PGCLCs were not purified for quantitative RT-PCR analysis, we cannot exclude a possibility that number of induced PGCLCs expressing *mil-1* and *Stella* was increased by knockdown of *Dnmt1*.

### The Bivalent Histone Modification of Non-PGC Genes Occurs in PGCs

**Figure 7:**
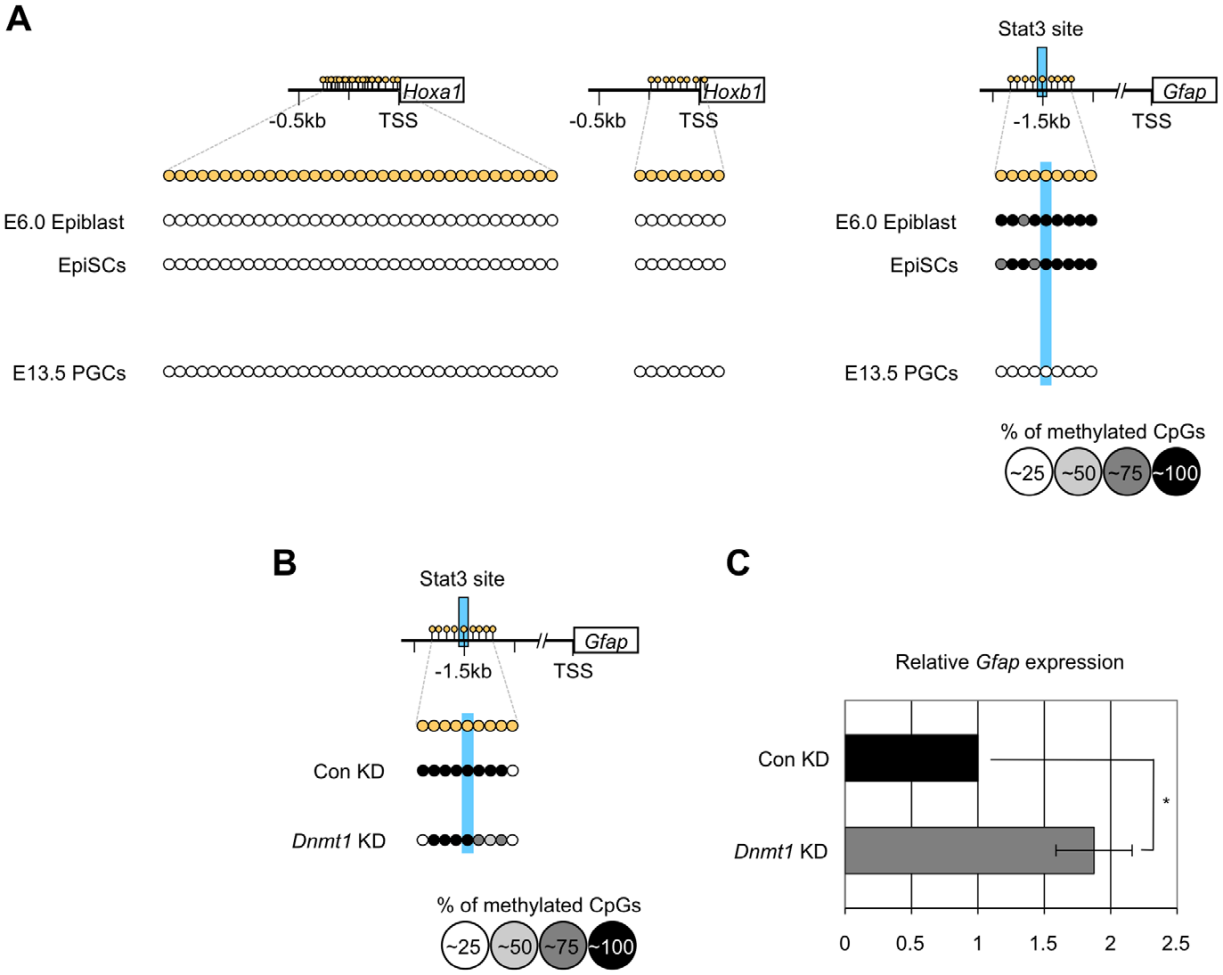
Repression of somatic gene expression does not depend on DNA methylation in PGCs. (A) Bisulfite sequencing analysis of the flanking regions of *Hoxa1*, *Hoxb1*, and *Gfap* was performed using epiblasts (E6.0), epiblast stem cells (EpiSCs), and PGCs (E13.5), showing hypomethylation in PGCs. (B) Bisulfite sequencing analysis of the regulatory region of *Gfap* and (C) quantitative RT-PCR analysis of the *Gfap* expression were performed using ES cells with or without *Dnmt1* knockdown treatment (*Dnmt1* KD/Con KD).

To examine whether DNA demethylation specifically activated the expression of PGC genes in PGCs, we examined DNA methylation status of the 5′-flanking regions of *Hoxa1*, *Hoxb1* and *Gfap* based on the bisulfite sequencing analysis (Figure 7, S5); the *Hox* genes represent somatic genes and *Gfap* is a neural cell-specific gene, whose expression was undetectable or very low in PGCs after E7.5, in epiblasts or in epiblast stem cells (EpiSCs) (data not shown). Surprisingly, we found that the flanking regions of the *Hox* genes were almost completely unmethylated in the proximal epiblasts at E6.0, in the gonadal PGCs at E13.5, and in EpiSCs (Figure 7A, S5). By contrast, the flanking region of *Gfap* gene was highly methylated in E6.0 epiblast and in EpiSCs, but became unmethylated in E13.5 PGC (Figure 7A, S5). These findings indicated that DNA methylation was not involved in repressing expression of *Hoxa1*, *Hoxb1* and *Gfap* in PGCs at E13.5. On the other hand, *Dnmt1* knockdown resulted in demethylation of the regulatory region of *Gfap* and upregulation of its expression in ES cells, as that of the PGC genes (Figure 7B, C, S5), suggesting that passive demethylation commonly results in upregulation of the PGC genes and of *Gfap* gene in ES cells, but the expression of *Gfap* in PGCs is likely repressed by additional epigenetic modification as shown below.

**Figure 8:**
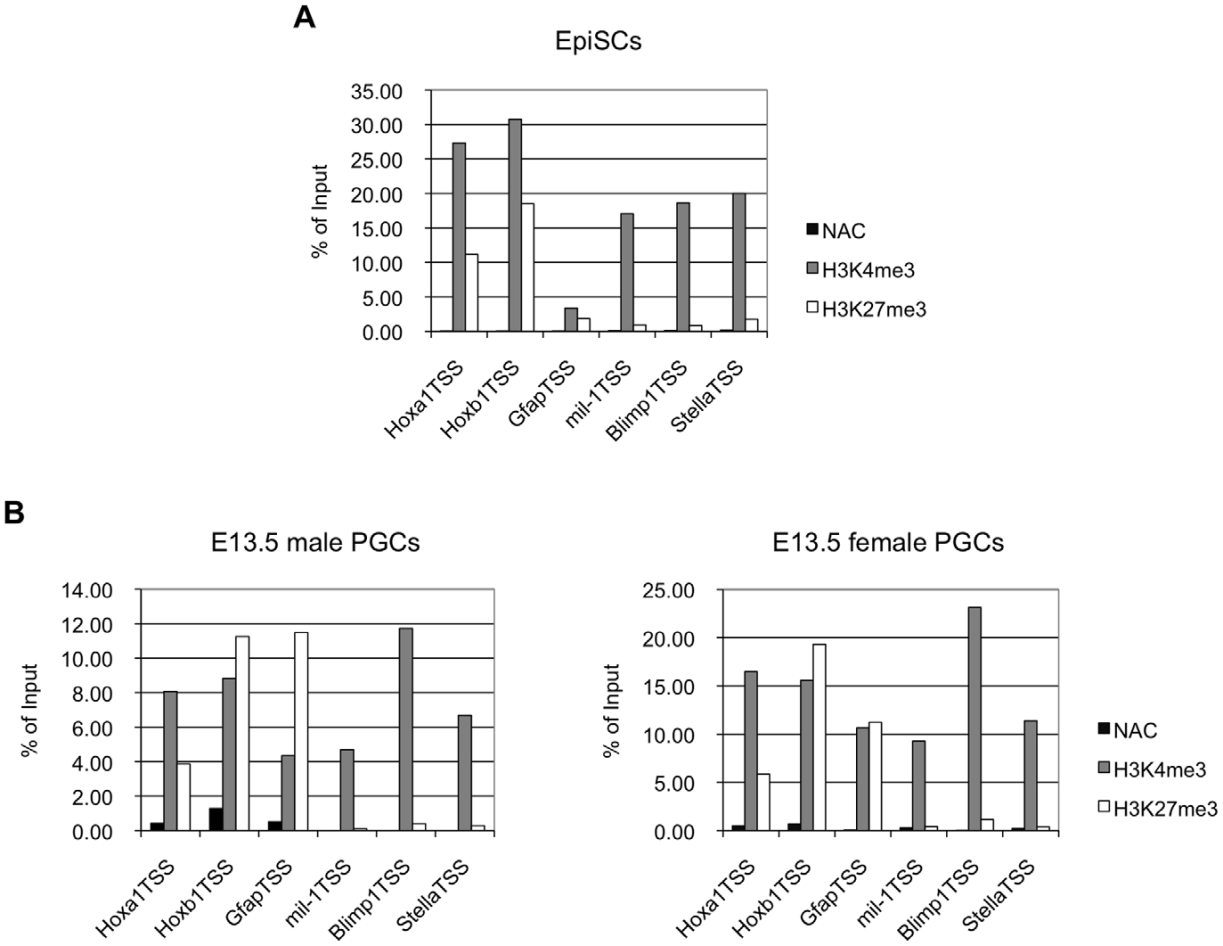
Bivalent histone modification on the somatic genes in PGCs. (A, B) ChIP analysis with the H3K4me3 or H3K27me3 antibodies for the promoter regions of somatic genes (*Hoxa1*, *Hoxb1*, and *Gfap*) and of the PGC-specific genes (*mil-1*, *Blimp1*, and *Stella*) was performed on EpiSCs (A), and male and female PGCs at E13.5 (B), showing the bivalent histone modification. Histogram represents ratios of the immuno-precipitated chromatin to the input chromatin, which was quantified by quantitative PCR analysis. Also shown are results using beads only as a no antibody control (NAC). Shown is a representative data from two independent experiments.

These results let us think about the notion of the bivalent histone modification [47]. In short, the flanking regions of somatic genes including *Hox* genes have non-methylated CpGs, but contain two reciprocal histone modifications i.e. Histone H3 Lysine 4 trimethylation (H3K4me3) (activating) and H3K27me3 (repressive), and their transcription is repressed or is poised for future activation in ES cells [47], [48]. We speculated that the bivalent histone modification also contribute to the repression of *Hox* genes and *Gfap* gene in PGCs. To address this hypothesis, we carried out ChIP analysis using antibodies against H3K4me3 or H3K27me3 to precipitate chromatin prepared from sorted male and female PGCs at E13.5 and from EpiSCs. The results clearly demonstrated that the regions flanking *Hoxa1* and *Hoxb1* were occupied by both H3K4me3 and H3K27me3 in both male and female PGCs as well as in EpiSCs, while the regions flanking *mil-1*, *Blimp1*, and *Stella* was predominantly occupied by H3K4me3 (Figure 8A, B). In the case of *Gfap*, its flanking region showed only low level binding of both H3K4me3 and H3K27me3 in EpiSCs, but showed the bivalent modification in PGCs. Together, these results suggested that the bivalent histone modification rather than DNA methylation contributed to silencing of those three somatic genes in PGCs, and that the bivalent modification of the *Hox* gene was established as early as at epiblast stage.

## Discussion

### DNA Demethylation is Involved in PGC-specific Gene Expression

Expression of genes preferentially expressed in PGCs, such as *mil-1*, *Blimp1*, and *Stella* initiates at around the time of PGC fate determination, and expression of those genes are further upregulated during PGC differentiation. Here, we demonstrated that regions flanking those genes that contain the ICE consensus element commonly underwent DNA demethylation that was synchronous with enhancement of co-expression of those PGC genes in differentiating PGCs (Figure 1, 2, 4, 1, S2). A recent genome-wide analysis also indicated that promoters of germline-specific genes were hypomethylated in PGCs [49], and our data further showed that genes preferentially expressed in PGCs at the time of specification onwards were demethylated in PGCs. When PGCs reach the genital ridges between E10.5 and E11.5, initial expression of other PGC genes, including *Mvh* and *Dazl*, is evident, and DNA demethylation of their flanking regions occurs concomitantly in PGCs, while these sequences remained hypermethylated in the surrounding somatic cells, where these genes were barely expressed [14] (Figure 4B, S2). In addition, Velasco *et al.* [50] demonstrated that the recruitment of a *de novo* DNA methyltransferase, Dnmt3b, via E2F6, a transcriptional repressor, mediated DNA methylation of putative regulatory regions and, consequently, silencing of several PGC-specific genes, including *Mvh*, in somatic cells. Taken together, these findings indicate that DNA demethylation regulates the expression of a number of genes specifically or preferentially expressed in PGCs throughout their development. If this hypothesis is correct, it is important to determine how the timing of DNA demethylation of differentially expressed PGC genes is controlled. Our *in silico* analysis indicated that ICEs were present in the flanking regions of *mil-1*, *Blimp1*, and *Stella*, but not in those of *Mvh* and *Dazl*; therefore, we hypothesized that binding of specific factors to the ICEs might induce coordinated DNA demethylation in the regions flanking genes that are induced at early stages of PGC development, whereas an independent mechanisms might control demethylation of other PGC genes. Such mechanisms may contribute to differential timings of DNA demethylation of PGC genes.

At the time of PGC specification, the PGC genes were highly upregulated (Figure 2), while demethylation of their flanking regions was moderate (Figure 1, 4, S1, S2), suggesting that DNA demethylation is not required for initial activation of the PGC genes, but additional events such as increased binding of gene-specific transcription factors may be necessary. Relatively small increases of the expression of the PGC genes in ES cells after knockdown of *Dnmt1* compared with upregulation of the specific genes in PGCs, also support this possibility. We examined possible importance of demethylation of some particular CpGs occurring in PGCs at E7.5 by using ES cells, and found that C to T base replacement in the CpGs at position 1, 6, 8–10 did not result in increased luciferase reporter activity in methylated condition (Figure S6). Instead, the luciferase reporter with the base replacement at position 1 caused decreasing activity even in unmethylated status. This suggests that the sequence around the CpG is important for the expression of *mil-1* irrespective of their methylation status, but we cannot currently conclude whether demethylation of the CpG has a function in activation of *mil-1* expression.

The flanking regions of the PGC genes underwent demethylation between E7.5 and E10.5 (Figure 1, 4, S1, S2), and their expression was further increased at this stage (Figure 2), suggesting that demethylation spreading throughout the regulatory region make a contribution to the enhancement of their expression. *Dnmt1* knockdown in PGCLCs resulted in increased expression of the PGC genes (Figure 6). Because PGCLCs in culture are closely correlated with *in vivo* PGCs [46], this result is consistent to the idea that DNA demethylation is involved in enhancement of PGC-specific gene expression.

In our bisulfite sequence analysis of the *mil-1* regulatory region, we found that the region near the transcription start site (TSS) was consistently hypomethylated in all cell types tested, including cells in which *mil-1* was barely expressed (Figure 1). Weber *et al.* [51] demonstrated that, for many germ line-specific genes in the human genome, non-CpG island regions (CpG ratio <0.48) near the TSSs are hypomethylated in somatic cells regardless of the level of expression. Therefore, our data suggested that the region near the *mil-1* TSS, which was a non-CpG island region (CpG ratio = 0.36) according to their criteria, was regulated in a similar fashion.

### Possible Involvement of Passive DNA Demethylation and Active DNA Demethylation

DNA demethylation mechanisms can be classified as passive (i.e., dependent on DNA replication) or active (i.e., independent of DNA replication). In cases of replication-dependent demethylation, a maintenance DNA methyltransferase, Dnmt1, is transiently downregulated in, at least a portion of PGCs at the time of PGC fate determination (E7.25); Dnmt1 is subsequently re-expressed by the time of PGC migration (E8.25) [4], [52]. Expression of Np95, a Dnmt1 cofactor, is also repressed in emerging PGCs (E7.0) [2]. Np95 recognizes hemi-methylated CpGs (i.e., those with only mother-strand methylation) and recruits Dnmt1 to these hemi-methylated sites [53]. Therefore, passive DNA demethylation may occur in regions flanking PGC genes in emerging PGCs owing to loss of Dnmt1- and/or Np95- recruitment [reviewed by 12]. We demonstrated that multiple PGC genes were hypomethylated and their expression was increased in *Dnmt1*-knockdown ES cells and PGCLCs (Figure 3, 5, 6, S3), and that *Gfap* was also hypomethylated in the *Dnmt1*-knockdown ES cells (Figure 7, S5). Those results suggest that passive DNA demethyaltion could occur at least in ES cells and PGCLCs, though it is currently unclear whether or not the passive mechanism is indeed involved in DNA demethylation in PGCs in vivo.

Furthermore, *de novo* expression of *Dnmt3a* and *3b* is specifically downregulated shortly before PGC fate determination at E6.75 [2] and maintained at low levels at E12.5 [52] in PGCs. Therefore, lack of Dnmt3a and Dnmt3b recruitment may contribute to maintenance of the hypomethylation of regions flanking PGC genes that resulted from prior demethylation, and this hypomethylation may promote PGC-specific gene expression. [reviewed by 12].

From E7.75 to E9.0, when PGCs migrate towards the forming genital ridges, most PGCs arrest in the G2 phase of the cell cycle [54]. Because the regulatory region of *mil-1* is still progressively demethylated in PGCs at E9.0 (Figure 1, S1), active DNA demethylation should also progress in PGCs during the G2 arrest. Candidate players in the active DNA demethylation of *mil-1*, such as growth arrest and DNA-damage-inducible protein 45α (*Gadd45α*) and a cytosine deaminase–*AID*, show relatively high expression in PGCs [2], [55]. Additionally, *Tet1,* a hydroxylase specific for methylated cytosines is also expressed in migrating PGCs (data not shown) and gonadal PGCs [56]. These findings indicate that *Gadd45α*, *AID*, and *Tet1* may be involved in active DNA demethylation of regions flanking PGC-specific genes in PGCs. Actually, AID was recently reported to play a role in DNA demethylation of region flanking *Dazl* [57].

### Mechanisms that Regulate Cell-type-specific gene Expression during PGC Development

Here, we showed that the CpGs in regions flanking *Hoxa1* and *Hoxb1*, two representative somatic genes not expressed in PGCs after E7.5, were consistently unmethylated in epiblasts and in PGCs at E13.5 (Figure 7A, S5), while the promoter regions of those genes showed the bivalent histone modification in PGCs as well as in EpiSCs (Figure 8), suggesting that these genes are poised for transcriptional activation in PGCs and in EpiSCs as in ES cells [47], [48]. Consisting with those results, *Ezh2*, an H3K27me3 methyltransferase, may be involved in the elevation of H3K27me3 because it is expressed in epiblasts and PGCs [4].

In the case of *Gfap* whose expression was undetectable in PGCs and in epiblast (data not shown), its regulatory region was hypermethylated in epiblast and in EpiSCs (Figure 7A, S5), and knockdown of *Dnmt1* in ES cells resulted in its increased expression (Figure 7C). In addition, the regulatory region became hypomethylated in PGCs (Figure 7A). The histone modification of this gene in EpiSCs was at low levels, but it showed the bivalent histone modification in PGCs as the *Hox* genes (Figure 8). The results suggest that the expression of *Gfap* is repressed by DNA methylation at least in ES cells. Although no functional evidence is available, we speculate that *Gfap* expression in PGCs is repressed by the bivalent histone modification as the *Hox* genes. By contrast, DNA demethylation is involved in enhancement of the expression of PGC genes as discussed above, and histone modifications of those genes seem to be active status, i.e., hypermethylated H3K4 and hypomethylated H3K27 (Figure 8).

During spermiogenesis, most histones are replaced with protamines, small basic proteins that form tightly packed DNA structures important for normal sperm functions. Surprisingly, a few nucleosomes are retained in human sperm nuclei, and these nucleosomes are significantly enriched at loci of somatic genes, including *HOX* gene clusters, and they carry bivalent histone modification [58]. Just after fertilization, paternal nuclei actively undergo DNA demethylation in genome-wide fashion [59], [60]. Hammoud *et al.* [58] found that no genes with bivalent histone modification in sperms were found in the gene-set that was highly expressed in 4-cell or 8-cell human embryos [61]. Hence, the bivalent histone modification in sperm nuclei may be a “safety devise” for appropriate gene expression even under the de-repressive conditions (i.e. genome-wide hypomethylated DNA state) of paternal nuclei in pre-implantation embryos. Although in-depth experimental evidence showing functional importance of the bivalent histone modification in PGCs is not so far available, the above mentioned study implies that bivalent histone modification also repress somatic genes in hypomethylated DNA state observed in PGCs. These epigenetic modifications may be coordinated to permit the PGC-specific genes to be expressed during germ cell development and to poise other somatic genes for future activation at later stages.

## Materials and Methods

### Ethics Statement

All the animal experiments were performed under the ethical guidelines of Tohoku University, and animal protocols were reviewed and approved by the Tohoku University Animal Studies Committee.

### Mice

MCH and C57BL/6J mice were purchased from Japan SLC, Inc. The *mil-1-green fluorescent protein (GFP)* [25], *Blimp1-GFP* [1], and *Oct4ΔPE-GFP* [22] transgenic mice were maintained in a C57BL/6J genetic background. These mice were kept and bred in the Animal Unit of the Institute of Development, Aging and Cancer (Tohoku University), an environmentally controlled and specific pathogen-free facility.

### Isolation of Epiblasts, PGCs, and Somatic Cells

Embryos were obtained from female MCH mice that were mated with male mice carrying a *mil-1-GFP*, *Blimp1-GFP*, or *Oct4ΔPE-GFP* transgene at appropriate embryonic days (noon of the day when a copulation plug was identified was designated as embryonic day 0.5 [E0.5]). E5.75–E13.5 embryos were collected and dissected in Dulbecco’s modified Eagle medium (DMEM; GIBCO) supplemented with 10% fetal bovine serum (FBS; BioWest). Epiblasts (E5.75–E6.75) were isolated from the extra-embryonic ectoderm and the surrounding visceral endoderm using fine forceps and a tungsten needle. The regions that contained primordial germ cells (PGCs) (i.e., the bases of allantoises at E7.25 to E7.5, the hindgut endoderm at E9.0, the dorsal mesenteries at E10.5, and the genital ridges at E13.5) were dissected from the embryos. Tissue fragments containing PGCs were trypsinized, PGCs (GFP-positive cells) at E7.25 to E7.5, and E9.0 were manually picked up and collected using a fine glass needle under a fluorescence stereomicroscope, or those at E10.5 and E13.5 were purified on EPICS ALTRA cell sorter (Beckman Coulter). GFP-negative cells were also collected to represent somatic cells at E10.5 and E13.5. For the analyses of *mil-1*, we used epiblasts (E5.75–E6.0) and PGCs (E7.25–E7.5) from *Blimp1-GFP* embryos, and PGCs (E9.0/E10.5/E13.5) from *Oct4ΔPE-GFP* embryos. For the analyses of the other genes *Blimp1*, *Stella*, *Hoxa1*, and *Hoxb1*, we used *mil-1-GFP* transgenic embryos at each developmental stage. We confirmed that all of the collected cells as PGCs expressed GFP.

### Mouse ES Cell Culture and *Dnmt1* Knockdown

Mouse feeder-free E14tg2a ES cells were cultured on gelatin-coated dishes in Glasgow Minimum Essential medium (GMEM; GIBCO) supplemented with 10% FBS (Moregate), 0.1 mM non-essential amino acid (NEAA; GIBCO), 1 mM sodium pyruvate (GIBCO), 100 µM β-mercaptoethanol (Sigma) and 1000 U/ml LIF (ESGRO; Chemicon).

To knock down Dnmt1 protein in the ES cells, 48 pmol of siRNA that targeted *Dnmt1* (QIAGEN, SI00189910; 5′-CTCGACCTGGTTTGATACTTA-3′) or AllStars negative control siRNA (QIAGEN, SI03650318) were transfected into ES cells using Lipofectamine RNAiMAX (Invitrogen). *Dnmt1*-knockdown and control knockdown ES cells were plated at 5,000 cells/well in 500 µl of medium in the 0.1% gelatin-coated 24-well culture plates (Falcon); cells were cultured in a conventional 5% CO_2_ incubator at 37°C for 72 h. Thereafter, knockdown ES cells were lysed for genomic DNA and total RNA extraction.

### Mouse EpiLCs-PGCLCs Induction and *Dnmt1* Knockdown

Mouse EpiLCs-PGCLCs induction was performed essentially as described previously [46]. Briefly, VR15 ES cells [62] were adapted to 2i (PD0325901; Wako, CHIR99021; AXON MEDCHEM) + LIF, feeder-free culture condition. The EpiLCs were induced by plating ES cells on a well coated with human plasma fibronectin (Millipore) in N2B27 medium (Stem Cell Sciences Ltd.) containing Activin A (R&D), bFGF (Sigma), and KSR (GIBCO). The PGCLCs were induced under a floating condition by plating EpiLCs in a well of a low-cell-binding U-bottom 96-well plate (NUNC) in a GMEM-based serum-free medium in the presence of the cytokines BMP4 (R&D), LIF (ESGRO; Chemicon), SCF (R&D), and EGF (Sigma). *Dnmt1* knockdown was performed to the EpiLCs at day 2 by forward transfection of the siRNA that targeted *Dnmt1* or of the AllStars negative control siRNA using Lipofectamine RNAiMAX. The EpiLCs were cultured in a conventional 5% CO_2_ incubator at 37°C for 24h, followed by the PGCLCs induction. Thereafter, the PGCLCs at day 2 were lysed for total RNA extraction.

### Mouse EpiSCs Culture

Mouse EpiSCs were cultured on gelatin-coated dishes with MEF (mouse embryonic fibroblasts) in DMEM/F-12 (GIBCO) supplemented with 20% KSR (GIBCO), 0.1 mM non-essential amino acid (NEAA; GIBCO), 1 mM sodium pyruvate (GIBCO), 0.2 mM L-glutamine (Sigma), 0.1 mM β-mercaptoethanol (Sigma), 10 ng ml^−1^ Activin A (R&D), and 5 ng ml^−1^ bFGF (Sigma).

### Bisulfite Sequencing Analysis

Genomic DNA was extracted from sorted PGCs and somatic cells (E10.5/E13.5), and knockdown ES cells, and EpiSCs using the QIAamp DNA Micro/Mini Kit (QIAGEN) and subjected to bisulfite conversion using the EpiTect Bisulfite Kit (QIAGEN) or the EZ DNA Methylation-Direct Kit (Zymo Research). We used about 5,000 and 100,000 cells of sorted PGCs and somatic cells at E10.5 and E13.5, respectively, and about 100,000 and 1000,000 ES cells and EpiSCs, respectively in each assay. Using the EZ DNA Methylation-Direct Kit, bisulfite reactions were performed directly on isolated epiblasts (E5.75–E6.75) and manually collected PGCs (E7.25–E7.5, E9.0) without DNA extraction. We usually used about 100 manually isolated epiblast cells (E5.75–E6.75) and 100 PGCs (E7.25–E7.5, E9.0) for each bisulfite reaction, and we confirmed that this number of ES cells gave essentially identical results with those by using large number (about 100,000) of ES cells (data not shown). Nested PCR was performed using AmpliTaq Gold DNA Polymerase (Applied Biosystems) or BIOTAQ HS DNA Polymerase (BIOLINE). The sequences of the PCR primers designed with MethPrimer (http://www.urogene.org/methprimer/index1.html) and the respective PCR conditions are listed in Table S1. The PCR products were gel-purified, subcloned into the pGEM-T Easy vector (Promega), and sequenced using an ABI PRISM 3100-Avant Genetic Analyzer (Applied Biosystems). Sequence data were analyzed with the QUantification tool for Methylation Analysis (QUMA; http://quma.cdb.riken.jp/top/index_j.html). We generally analyzed about 10 to 20 clones to confirm the sequence of each region. In the analyses of the sorted cells and the ES cells, data were obtained from one experiment or two independent experiments. In the analyses of a small number of manually acquired cells (i.e. epiblasts and PGCs), the data were obtained from two to four independent experiments.

### Quantitative RT-PCR Analysis

Total RNA were extracted from each embryonic sample, EpiSCs, EpiLCs, or from knockdown ES cells/PGCLCs and purified using the RNeasy Micro Kit (QIAGEN) or the RNeasy Plus Mini Kit (QIAGEN). Total RNA were reverse transcribed by Superscript β (Invitrogen), and the first-strand cDNAs were used for quantitative RT-PCR analysis with the EXPRESS qPCR SuperMix (Invitrogen) and the TaqMan Gene Expression Assay (Applied Biosystems) according to the manufacturer’s instructions. PCR signals were detected using ABI PRISM 7000 (Applied Biosystems).

### Luciferase Reporter Assay

The 3.0 kbp regulatory sequence of *mil-1* was cloned into the CpG-free pCpGL-basic luciferase vector [63] by ligation. Mutated plasmids that have C to T base replacement in some CpGs in the 3.0 kbp sequence by site-directed mutagenesis using the QuikChange Lightning Multi Site-Directed Mutagenesis Kit (Agilent Technologies) were also constructed. The sequences of the mutagenesis primers are listed in Table S3. Luciferase reporter constructs were either mock-treated or methylated *in vitro* with SssI methylase for 4 h at 37°C and purified with the QIAquick Purification Kit (QIAGEN). 500 ng of each reporter plasmid and 50 ng of *Renilla* phRL-TK control vector (Promega) were co-transfected into ES cells using Lipofectamine LTX (Invitrogen). Cells were lysed after 48 h, and assayed for firefly and *Renilla* luciferase activities using the Dual-Luciferase Reporter Assay System (Promega) on a Lumat LB 9507 (Berthold). Firefly luciferase activity of individual transfections was normalized against *Renilla* luciferase activity.

### Chromatin Immunoprecipitation (ChIP) Analysis

About 300,000 cells of sorted male or female PGCs at E13.5, and about 1000,000 cells of EpiSCs were cross-linked by directly adding 37% formaldehyde to the cell suspension to a final concentration of 1% and were incubated at room temperature with gentle inverting for 10 min, then the reaction was quenched by adding 1.25 M glycine to 200 mM of final concentration. Cross-linked cells were washed with ice-cold phosphate saline buffer (PBS) and centrifuged, then cell pellets were snap-frozen in liquid Nitrogen to be stored at −80°C until use.

Cell pellets were lysed in 100 µl SDS Lysis Buffer (50 mM Tris-HCl [pH 8.0], 10 mM EDTA and 1% SDS), and the lysates were suspended in 400 µl ice-cold ChIP Dilution Buffer (50 mM Tris-HCl [pH 8.0], 167 mM NaCl, 1.1% Triton X-100, 0.11% sodium deoxycholate and protease inhibitor cocktail [complete; Roche]). The chromatin was fragmented by sonication (Branson Sonifier 250A with microtip; 30 sec. pulses with 30 sec. rests; total processing time of 12 min; output level 4). After centrifugation to remove insoluble materials, the fragmented chromatin in the supernatant was diluted up to 1 ml with ice-cold ChIP Dilution Buffer, and was dispensed into 300 µl (three tubes) for each immunoprecipitation and 30 µl (one tube) as ‘Input’ which was stored at 4°C until DNA purification. 200 µl of ice-cold RIPA-150 mM NaCl (50 mM Tris-HCl [pH 8.0], 150 mM NaCl, 1 mM EDTA, 0.1% SDS, 1% Triton X-100, 0.1% sodium deoxycholate and protease inhibitor cocktail) was added to the fragmented chromatin up to 500 µl.

H3K4me3 antibody was purchased from Abcam (catalog no. ab8580); H3K27me3 antibody was purchased from Upstate (catalog no. 07-449). For each immunoprecipitation, 50 µl Dynabeads Protein G (Invitrogen) were washed with PBS and incubated with 10 µg of the indicated antibody in 500 µl RIPA-150 mM NaCl overnight at 4°C with rotation, and washed twice with 1 ml of ice-cold RIPA-150 mM NaCl. An aliquot of the fragmented chromatin (500 µl) was incubated with the antibody-bound Dynabeads overnight at 4°C with rotation. The beads were washed sequentially with 1 ml of ice-cold RIPA-150 mM NaCl, 1 ml of ice-cold RIPA-500 mM NaCl (50 mM Tris-HCl [pH 8.0], 500 mM NaCl, 1 mM EDTA, 0.1% SDS, 1% Triton X-100 and 0.1% sodium deoxycholate), and twice with 1 ml of ice-cold TE (10 mM Tris-HCl [pH 8.0] and 1 mM EDTA). After removing TE, the beads were mixed with 200 µl Direct Elution Buffer (10 mM Tris-HCl [pH 8.0], 300 mM NaCl, 5 mM EDTA and 0.5% SDS) and incubated overnight at 65°C to reverse cross-linking. After this, the same procedures were also performed on the ‘Input’ (30 µl), which was mixed with 170 µl Direct Elution Buffer and 1.3 µl 10% SDS. Samples were then treated with RNaseA (Roche; 5 µg/ml; 37°C; 30 min) and proteinase K (Sigma; 500 µg/ml; 55°C; 2–3 h). DNA was cleaned up using QIAquick PCR Purification Kit (QIAGEN).

The enrichment of specific regions in each of the immunoprecipitated DNA was analyzed by quantitative PCR with the *Power* SYBR Green PCR Master Mix (Applied Biosystems) according to the manufacturer’s instruction, and was expressed as percentages of Input DNA, which was determined using the 2^−ddCt^ method as outlined in the Applied Biosystems protocol “User Bulletin #2”. PCR signals were detected by ABI PRISM 7000 (Applied Biosystems). The sequences of the PCR primers are listed with their PCR conditions in Table S2. The data were obtained from two independent experiments.

### Statistical Analysis

Data were analyzed using the Student’s *t*-Test. *P-*values <0.05 were considered statistically significant.

## Supporting Information

Figure S1 **Data of individual clones for bisulfite sequencing analysis of the regulatory region of** ***mil-1*** **in epiblasts, PGCs and somatic cells from embryos at each embryonic day (E).** Closed circles correspond to methylated CpGs, while open circles correspond to unmethylated ones. Each sequence data was obtained from three independently isolated cells of embryos at E6.0, E7.5, and E9.0, and from a single sample of purified PGCs and surrounding somatic cells of embryos at E10.5 and E13.5. [Related to Figure 1]
(TIF)

Figure S2 **Data of individual clones for bisulfite sequencing analysis of the flanking regions of** ***Blimp1*** **,** ***Stella*** **, and** ***Dazl*** **in epiblasts, PGCs from embryos.** Each sequence data was obtained from two to four independently isolated cells of embryos at E6.0, E6.75, and E7.5, and from a single sample of purified PGCs at E10.5. [Related to Figure 4]
(TIF)

Figure S3 **Data of individual clones for bisulfite sequencing analysis of the flanking regions of** ***mil-1*** **,** ***Blimp1*** **, and** ***Stella*** **on ES cells with or without** ***Dnmt1*** **knockdown treatment (** ***Dnmt1*** **KD/Con KD).** Each sequence data was obtained from a single sample. [Related to Figure 3 and Figure 5]
(TIF)

Figure S4 **Deficiency of** ***Dnmt1*** **does not affect the expression of pluripotency-related** ***Oct4*** **and** ***Nanog*** **in ES cells.** Quantitative RT-PCR analysis of *Oct4* and *Nanog* expression was performed using ES cells with or without *Dnmt1* knockdown treatment (*Dnmt1* KD/Con KD). [Related to Figure 3 and Figure 5]
(TIF)

Figure S5 (A) Data of individual clones for bisulfite sequencing analysis of the flanking regions of *Hoxa1*, *Hoxb1*, and *Gfap* in epiblasts, EpiSCs, and PGCs. (B) Data of individual clones for bisulfite sequencing analysis of the regulatory region of *Gfap* in ES cells with or without *Dnmt1* knockdown treatment (*Dnmt1* KD/Con KD). Each sequence data was obtained from a single sample of EpiSCs, of purified PGC at E13.5 and of ES cells with Con KD or with *Dnmt1* KD. [Related to Figure 7]
(TIF)

Figure S6 (A, B) Luciferase activities from the luciferase reporter vectors of the *in vitro* methylated or unmethylated regulatory region of *mil-1* with C to T replacement of some CpG sites in ES cells (B). Number within parenthesis indicates positions of C to T replacement shown in (A). Luciferase activity was normalized against the activity of a co-transfected *Renilla* construct. The data were obtained from four independent experiments. *p<0.05. Error bars represent SEM.
(TIF)

Table S1 **Primers used for bisulfite sequence analyses.**
(XLSX)

Table S2 **Primers used for quantitative PCR analyses of ChIP DNA.**
(XLSX)

Table S3 **Primers used for site-directed mutagenesis of regulatory sequence of** ***mil-1*** **for the luciferase reporter assay.** Capital ‘T’s indicate positions of C to T replacement of CpG sites.
(XLSX)
