# Supplementary figures and images for: Implication of DNA Demethylation and Bivalent Histone Modification for Selective Gene Regulation in Mouse Primordial Germ Cells
Source: PLoS One. 2012 Sep 28;7(9):e46036. doi: 10.1371/journal.pone.0046036 (PMC3461056; doi:10.1371/journal.pone.0046036)

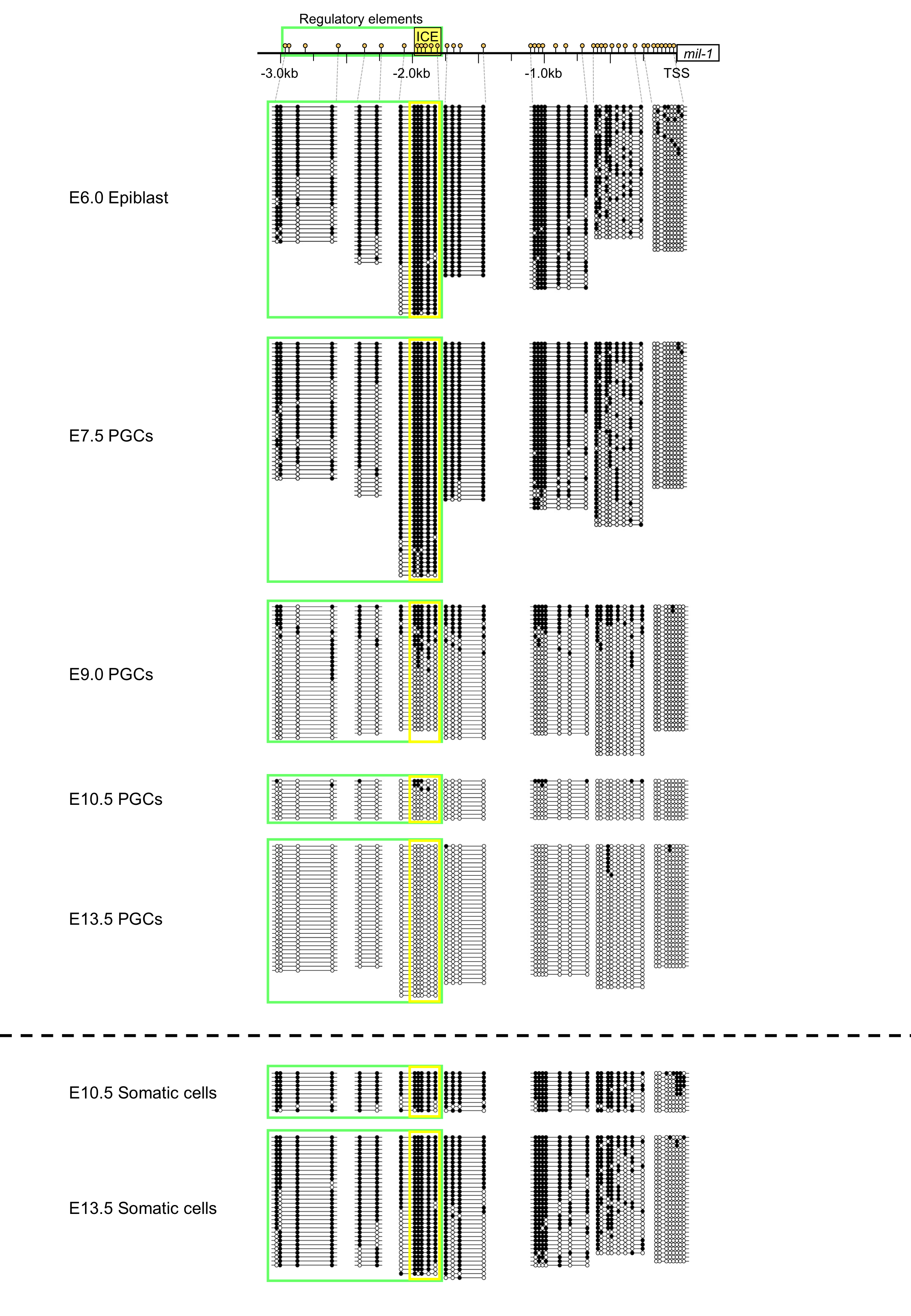

Supplement: Figure S1 — Data of individual clones for bisulfite sequencing analysis of the regulatory region of mil-1 in epiblasts, PGCs and somatic cells from embryos at each embryonic day (E). Closed circles correspond to methylated CpGs, while open circles correspond to unmethylated ones. Each sequence data was obtained from three independently isolated cells of embryos at E6.0, E7.5, and E9.0, and from a single sample of purified PGCs and surrounding somatic cells of embryos at E10.5 and E13.5. [Related to Figure 1] (TIF) [file pone.0046036.s001.tif]

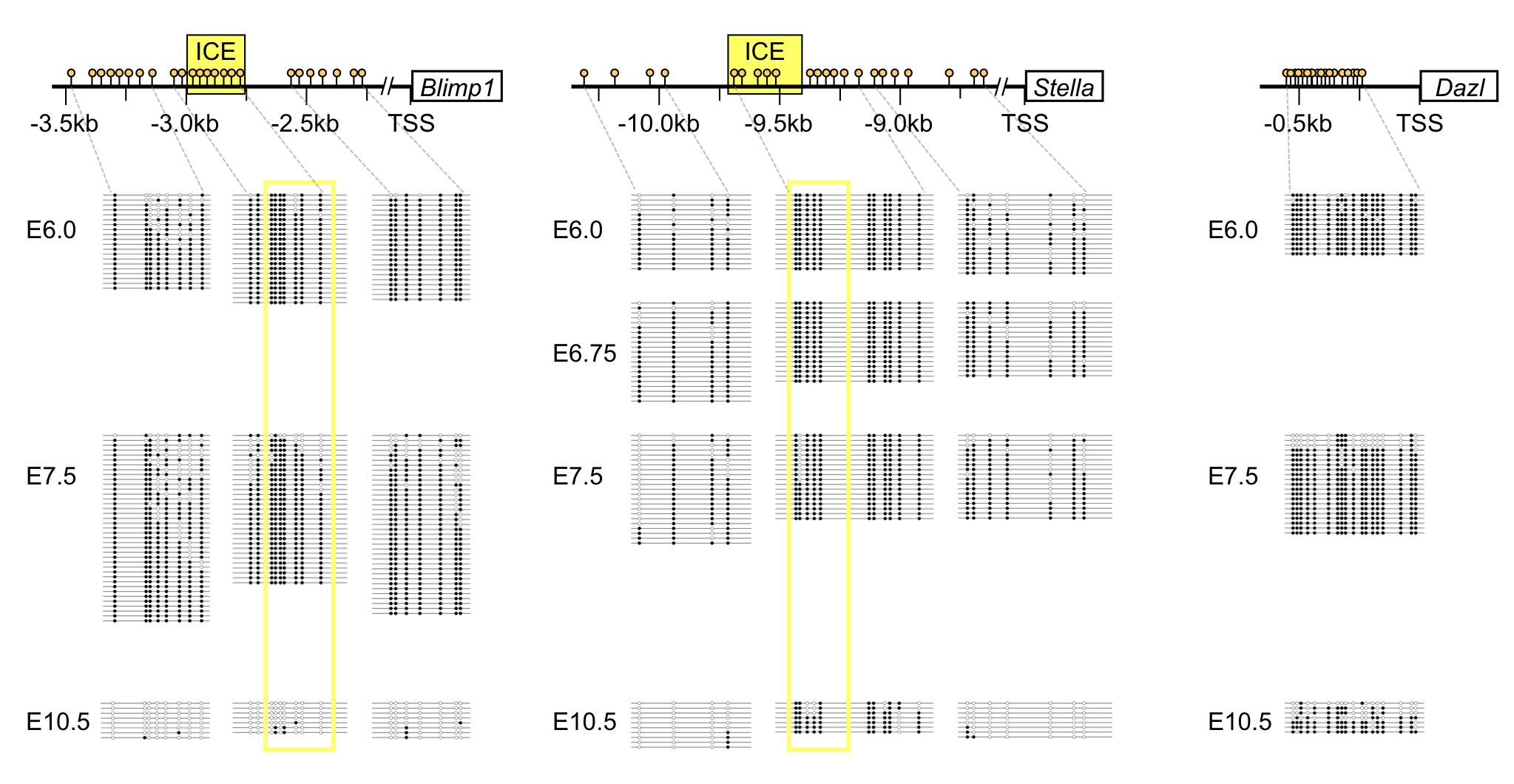

Supplement: Figure S2 — Data of individual clones for bisulfite sequencing analysis of the flanking regions of Blimp1 , Stella , and Dazl in epiblasts, PGCs from embryos. Each sequence data was obtained from two to four independently isolated cells of embryos at E6.0, E6.75, and E7.5, and from a single sample of purified PGCs at E10.5. [Related to Figure 4] (TIF) [file pone.0046036.s002.tif]

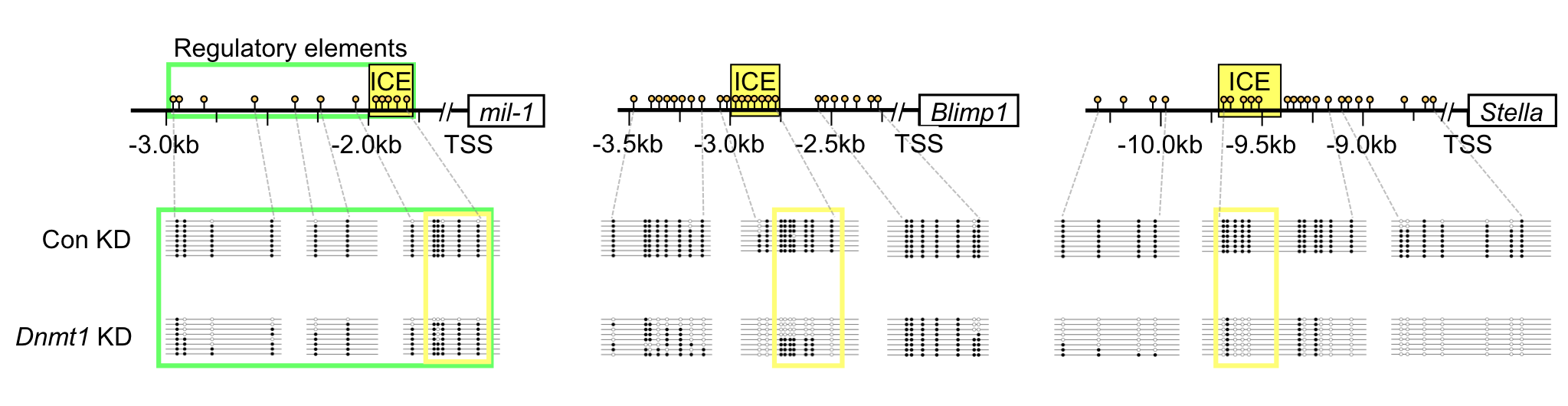

Supplement: Figure S3 — Data of individual clones for bisulfite sequencing analysis of the flanking regions of mil-1 , Blimp1 , and Stella on ES cells with or without Dnmt1 knockdown treatment ( Dnmt1 KD/Con KD). Each sequence data was obtained from a single sample. [Related to Figure 3 and Figure 5] (TIF) [file pone.0046036.s003.tif]

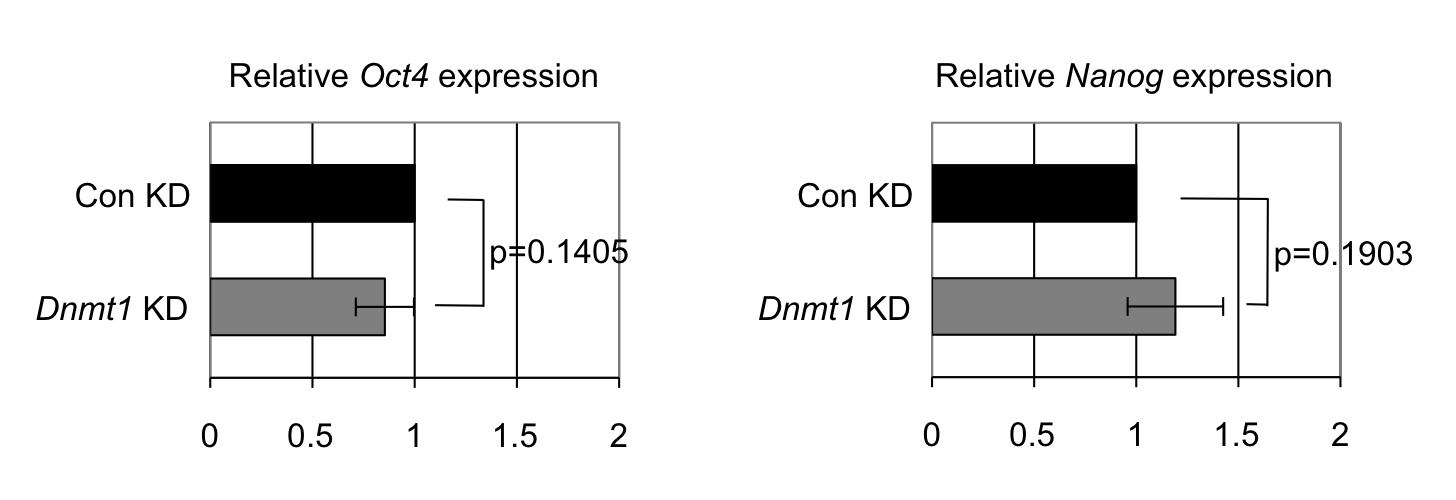

Supplement: Figure S4 — Deficiency of Dnmt1 does not affect the expression of pluripotency-related Oct4 and Nanog in ES cells. Quantitative RT-PCR analysis of Oct4 and Nanog expression was performed using ES cells with or without Dnmt1 knockdown treatment (Dnmt1 KD/Con KD). [Related to Figure 3 and Figure 5] (TIF) [file pone.0046036.s004.tif]

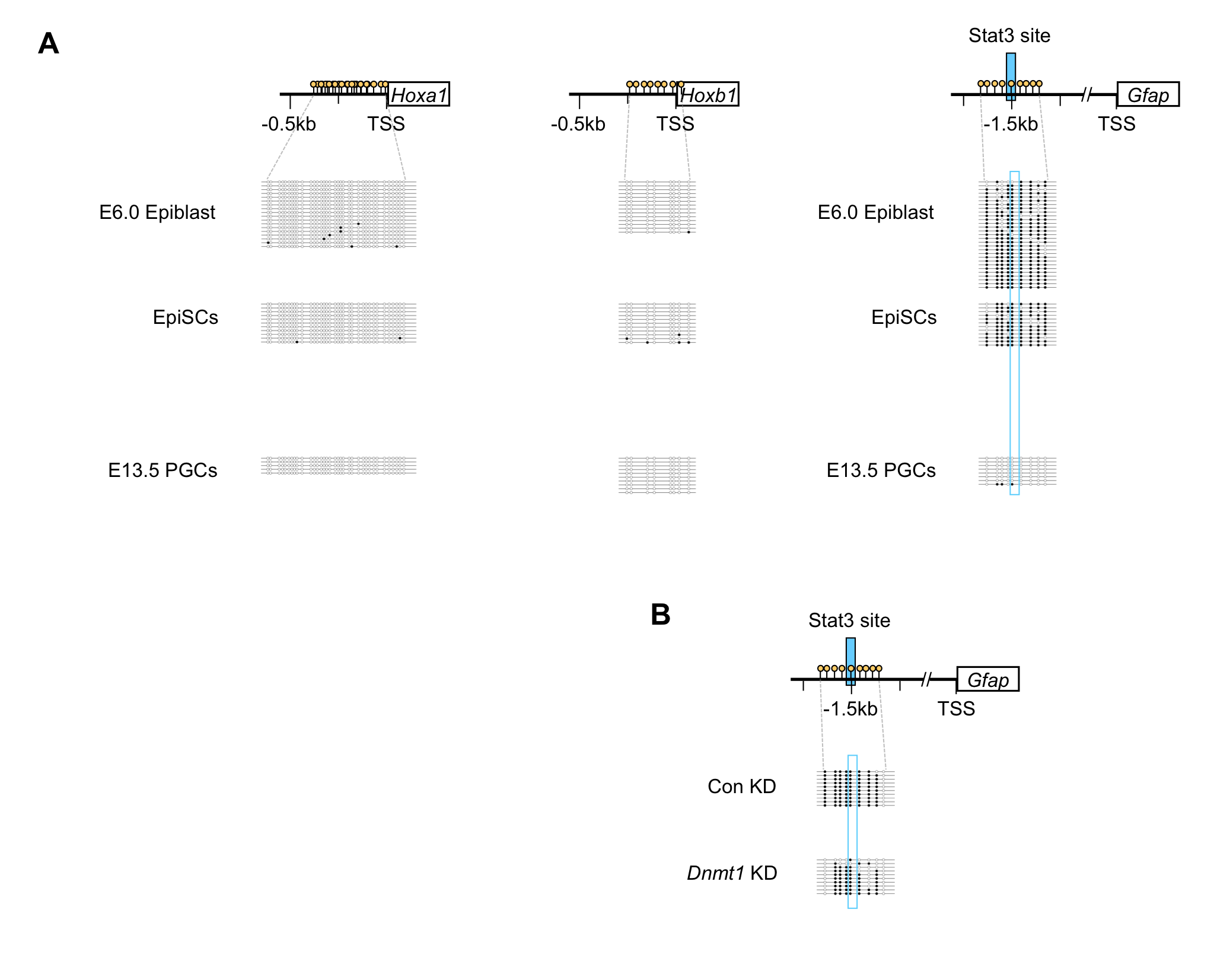

Supplement: Figure S5 — (A) Data of individual clones for bisulfite sequencing analysis of the flanking regions of Hoxa1, Hoxb1, and Gfap in epiblasts, EpiSCs, and PGCs. (B) Data of individual clones for bisulfite sequencing analysis of the regulatory region of Gfap in ES cells with or without Dnmt1 knockdown treatment (Dnmt1 KD/Con KD). Each sequence data was obtained from a single sample of EpiSCs, of purified PGC at E13.5 and of ES cells with Con KD or with Dnmt1 KD. [Related to Figure 7] (TIF) [file pone.0046036.s005.tif]

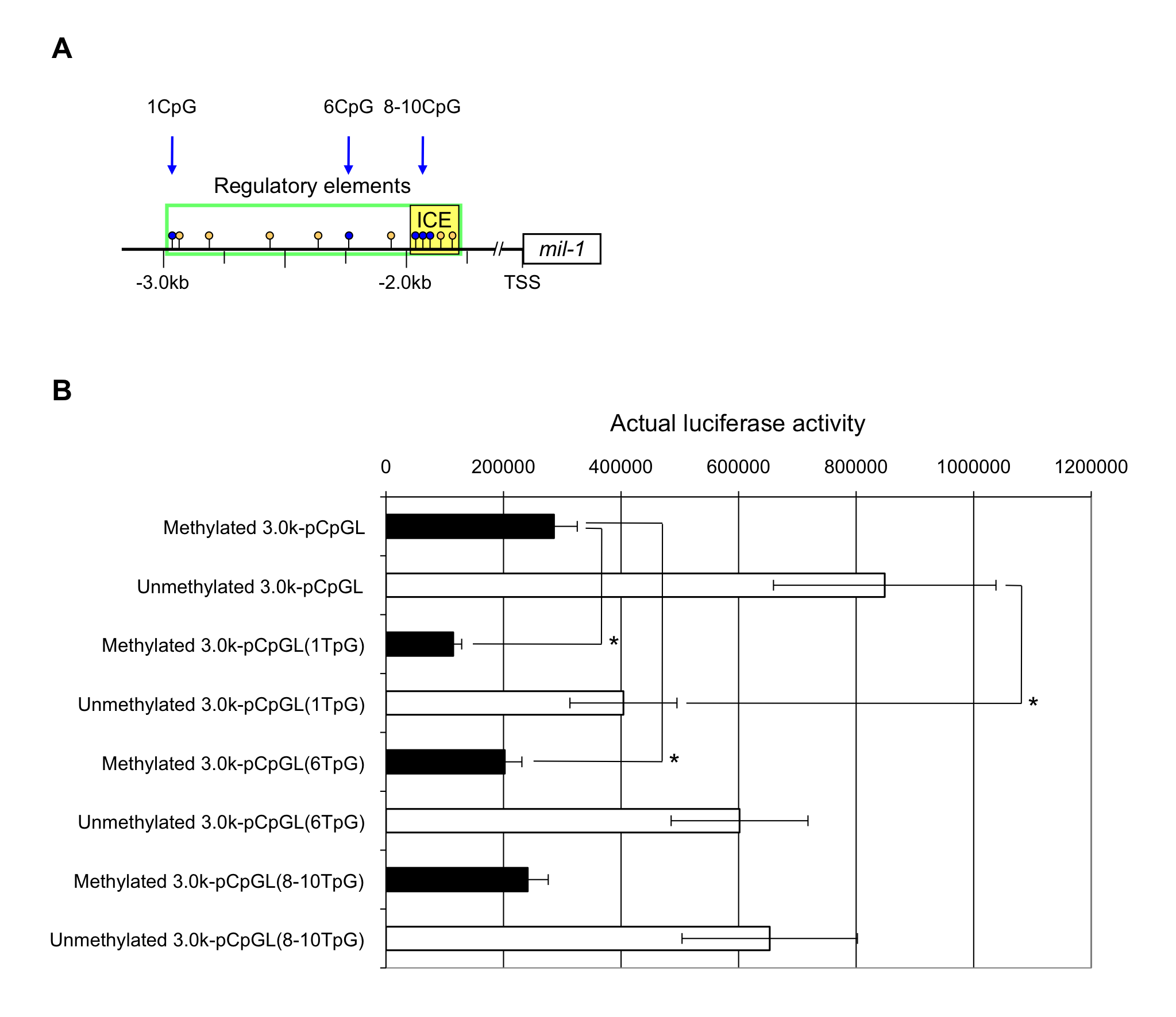

Supplement: Figure S6 — (A, B) Luciferase activities from the luciferase reporter vectors of the in vitro methylated or unmethylated regulatory region of mil-1 with C to T replacement of some CpG sites in ES cells (B). Number within parenthesis indicates positions of C to T replacement shown in (A). Luciferase activity was normalized against the activity of a co-transfected Renilla construct. The data were obtained from four independent experiments. *p<0.05. Error bars represent SEM. (TIF) [file pone.0046036.s006.tif]
